# miR-126-3p Inhibits Thyroid Cancer Cell Growth and Metastasis, and Is Associated with Aggressive Thyroid Cancer

**DOI:** 10.1371/journal.pone.0130496

**Published:** 2015-08-05

**Authors:** Yin Xiong, Shweta Kotian, Martha A. Zeiger, Lisa Zhang, Electron Kebebew

**Affiliations:** 1 Endocrine Oncology Branch, Center for Cancer Research, National Cancer Institute, Bethesda, Maryland, United States of America; 2 Department of Surgery, Johns Hopkins University, Baltimore, Maryland, United States of America; Sun Yat-sen University Medical School, CHINA

## Abstract

**Background:**

Previous studies have shown that microRNAs are dysregulated in thyroid cancer and play important roles in the post-transcriptional regulation of target oncogenes and/or tumor suppressor genes.

**Methodology/Principal Findings:**

We studied the function of miR-126-3p in thyroid cancer cells, and as a marker of disease aggressiveness. We found that miR-126-3p expression was significantly lower in larger tumors, in tumor samples with extrathyroidal invasion, and in higher risk group thyroid cancer in 496 papillary thyroid cancer samples from The Cancer Genome Atlas study cohort. In an independent sample set, lower miR-126-3p expression was observed in follicular thyroid cancers (which have capsular and angioinvasion) as compared to follicular adenomas. Mechanistically, ectopic overexpression of miR-126-3p significantly inhibited thyroid cancer cell proliferation, *in vitro* (p<0.01) and *in vivo* (p<0.01), colony formation (p<0.01), tumor spheroid formation (p<0.05), cellular migration (p<0.05), VEGF secretion and endothelial tube formation, and lung metastasis *in vivo*. We found 14 predicted target genes, which were significantly altered upon miR-126-3p transfection in thyroid cancer cells, and which are involved in cancer biology. Of these 14 genes, *SLC7A5* and *ADAM9* were confirmed to be inhibited by miR-126-3p overexpression and to be direct targets of miR-136-3p.

**Conclusions/Significance:**

To our knowledge, this is the first study to demonstrate that miR-126-3p has a tumor-suppressive function in thyroid cancer cells, and is associated with aggressive disease phenotype.

## Introduction

Thyroid cancer is the most common endocrine cancer and one of the most rapidly growing cancer diagnoses in the United States [1,2]. Thyroid cancers originate from parafollicular cells (medullary) and follicular cells (non-medullary), which account for over 95% of all thyroid cancer cases and are classified into four major histologic groups: follicular thyroid cancer (FTC), papillary thyroid cancer (PTC), anaplastic thyroid cancer (ATC), and Hürthle cell carcinoma (HCC).

MicroRNAs (miRNAs) are small, noncoding RNAs that are approximately 21 nucleotides long and regulate gene expression [3,4]. miRNAs play a significant role in tumorigenesis and show remarkable tissue specificity, and miRNAs have also been found to be good cancer biomarkers [5]. Previous studies have shown that several miRNAs are dysregulated in thyroid cancers originating from follicular cells [6–8]. In our previous study, we found that the expression of miR-126-3p was downregulated in malignant thyroid tumor samples as compared to benign thyroid tumor samples [9,10]. Downregulated miR-126-3p expression was observed in FTC and HCC, which are only histologically distinguishable from follicular or Hürthle cell adenomas when capsular invasion and/or angioinvasion are present. The role of miR-126-3p in thyroid cancer has not been studied previously, but our expression analysis in thyroid cancer samples suggests that the loss of miR-126-3p may be associated with thyroid cancer progression, and that it may function as a tumor suppressor.

In the present study, we tested the hypothesis that miR-126-3p is a tumor suppressor and is associated with disease phenotype. We determined the function of miR-126-3p in thyroid cancer cells, using both *in vitro* and *in vivo* models. We found that overexpression of miR-126-3p significantly inhibited thyroid cancer cell proliferation, colony formation, tumor spheroid formation, migration, VEGF secretion and HUVEC tube formation, and lung metastases *in vivo*. Furthermore, we found that miR-126-3p regulates the expression of many cancer-related genes, including those encoding solute carrier family 7 member 5 (*SLC7A5*) and a disintegrin and metalloproteinase domain-containing protein 9 (*ADAM9*), and that it directly targets these genes. Lastly, miR-126-3p expression was found to be associated with disease aggressiveness.

## Methods

The Animal Care and Use Committee of the National Cancer Institute, National Institutes of Health approved the animal study protocol. When mice reached humane euthanasia criteria, the mice were imaged, and euthanized by CO2 inhalation. The study was approved by the Office for Human Research Protections and patient information was collected prospectively under an institutional review board-approved protocol at the National Institutes of Health Clinical Center (Bethesda, Maryland) after obtaining written informed consent.

### Human thyroid tumor samples, cell lines and culture conditions

Human thyroid tissue was obtained at the time of tumor removal, immediately snap-frozen and stored at −80°C. Serial tissue sections were used for RNA extraction and stained with hematoxylin and eosin to confirm the diagnosis and that the tumor cell content was 80% or higher. The study was approved by the Office for Human Research Protections and patient information was collected prospectively under an institutional review board-approved protocol at the National Institutes of Health after obtaining written informed consent.

Human thyroid cancer cell lines XTC-1 (HCC), FTC-133 (FTC), and TPC-1 (PTC) were maintained in DMEM with 4,500 mg/L of D-glucose and L-glutamine, and 110 mg/L of sodium pyruvate, supplemented with 10% fetal bovine serum (FBS), thyroid-stimulating hormone (10 mU/mL), penicillin (10,000 U/mL), streptomycin (10,000 U/mL), Fungizone (250 mg/mL), and insulin (10 μg/mL) in a standard humidified incubator at 37°C in a 5% CO_2_ and 95% O_2_ atmosphere. FTC-133-Luc2 cells were generated by transfecting FTC-133 cells with a luciferase-expressing vector, and the cells were cultured in the same medium with G418 at a concentration of 200 μg / mL [11]. The cell lines were authenticated using short tandem repeat profiling.

### miRNA transfection

A miRNA mimic for hsa-miR-126-3p (miRNA mimic, Assay ID: MC12841; Applied Biosystems, Foster City, CA) was transfected into cells at a concentration of 25 nM using Lipofectamine RNAiMAX (Invitrogen, Carlsbad, CA), following the manufacturer’s protocol. An oligonucleotide not representing any known miRNA (miRNA Mimic Negative Control #1; Applied Biosystems) was used as a negative control.

### RNA isolation and quantitative real-time RT-PCR

Total RNA was isolated from the cell lines and tissue samples using the TRIzol reagent (Invitrogen, Carlsbad, CA). The TaqMan MicroRNA Assay (Applied Biosystems, Carlsbad, CA) was used to measure miR-126-3p expression level (hsa-miR-126-3p, Assay ID: 002228). Total RNA was reverse transcribed with a miRNA-specific primer, followed by real-time PCR using TaqMan probes. U6 was used as an endogenous control. The relative amounts of *ADAM9* and *SLC7A5* mRNAs were determined using the TaqMan Assay (Applied Biosystems) on an ABI 7900 HT system; human *GAPDH* was used as an endogenous control. The ΔΔ Ct method was used to calculate expression levels.

### Western blot

Whole-cell lysate was prepared with RIPA buffer (Thermo Fisher Scientific, Rockford, IL) and was used for ADAM9 protein detection by Western blot using a rabbit polyclonal anti-ADAM9 antibody (1:1000 dilution; Cell Signaling Technology, Inc., Danvers, MA) and for SLC7A5 protein detection by Western blot using a rabbit polyclonal anti-SLC7A5 antibody (1:500 dilution; Cell Signaling Technology, Inc., Danvers, MA). GAPDH protein, a control, was detected by using a mouse monoclonal anti-GAPDH (#0411) antibody (Santa Cruz Biotechnology, Santa Cruz, CA).

### Proliferation assay

Cell proliferation was determined using the CyQUANT Cell Proliferation Assay (Invitrogen), according to the manufacturer’s protocol. The fluorescence intensity was measured using a fluorescence microplate reader (Molecular Devices, Sunnyvale, CA), with excitation at 485 nm and emission detection at 538 nm.

### Migration assay

Cellular migration was measured using a BD Chamber (Catalog #354578, BD Biosciences, Bedford, MA), according to the manufacturer’s instructions. Cell culture medium with 10% FBS was used as a chemoattractant in the lower well of the Boyden chamber. Thyroid cancer cells were seeded in the upper compartment of the chamber in serum-free medium (4 × 10^4^ cells per well). After incubation at 37°C in 5% CO_2_ for 22 hours, the non-migrating cells were removed from the upper surface, and the cells that had migrated through the membrane to the lower surface were stained with the Diff-Quik Stain Set (Siemens Healthcare Diagnostics, Inc., Newark, DE). Images were taken from the membrane of each insert under a microscope (50× magnification) using a digital camera. The images were viewed on the computer screen and the cells in five fields of each insert were counted.

### Spheroid culture

Two days after miRNA transfection, cells were trypsinized, counted, resuspended in culture media, and plated in an Ultra Low Cluster plate (Costar, Corning, NY) at 3.5 × 10^4^ cells per well. The plates were cultured at 37°C in 5% CO_2_, and the medium was changed every 2 to 3 days. After 2 weeks of culture, cells were stained with crystal violet and photographed under a microscope. The total area occupied by spheroids within an image was measured by circumscribing the perimeter of each spheroid, marking the entire area, and calculating the pixel numbers using ImageJ software (National Institutes of Health, Bethesda, MD, USA).

### Soft agar assay for colony formation

Three days after miRNA transfection, FTC-133 cells were trypsinized, counted, and resuspended in culture media. Two-layered soft agar assays were performed in six-well plates. The bottom layer of agar (2 mL/well) contained 0.6% agar (Difco agar noble; BD Diagnostics, Sparks, MD) in Ham’s F-12 medium, supplemented with 10% FBS, penicillin (100 U/mL), streptomycin (10,000 U/mL), and Fungizone (250 ng/mL). Thirty thousand cells were mixed with 1 mL of upper agar solution (0.35% agar in culture media). After 30 minutes, 1 mL of culture media was added to each well. The plates were cultured at 37°C in 5% CO_2_, and the media were changed twice a week. After two weeks of culture, cell colonies were stained with crystal violet and examined under a microscope. Colony counting was performed in three different fields.

### Tumor xenograft studies

FTC-133 cells containing luciferase reporter gene *luc2* were transfected with miR-126-3p or miR-NC and inoculated subcutaneously (10^6^ viable cells) into the left and right flanks of athymic nude mice. Tumors were measured with calipers at different time points, and volumes were calculated as length × width × height. Autopsy tumor samples were photographed to document gross morphology, and then samples were weighed. FTC-133-*luc2* cells (7.5 × 10^5^) transfected with miR-126-3p and miR-NC were injected into athymic nude mice via the tail vein, and the mice were imaged weekly using a Xenogen IVIS 100 system.

### Wound healing assay

Thyroid cancer cell migration was assessed using a scratch wound healing assay [12] in which 150,000 cells were transfected with miRNAs, plated in six-well plates, and allowed to attach and grow for 44 hours (miRNAs). Next, three vertical wounds were made with a sterile 10-μl pipette tip, and then a horizontal line was made across the three lines so that cells could be observed at the same point. The cells were inspected every 12 hours and width measurements taken up to 24 hours.

### VEGF ELISA

Culture media supernatant was collected 72 hours after transfection of thyroid cancer cells with miR-NC or miR-126-3p. VEGF levels were measured using the Human VEGF Quantikine ELISA kit (R & D Systems, Minneapolis, MN). VEGF levels were normalized to total protein levels using the Pierce BCA Protein Assay kit (Pierce Biotechnology, Rockford, IL).

### Immunohistochemistry

Paraffin embedded lung tissue sections from mice injected with FTC-133-Luc2 cells transfected with either miR-NC or miR-126-3p were de-paraffinized and rehydrated. Sections were then treated with anti-VEGF (ab46154, Abcam, Cambridge, MA). Slides were scanned using a ScanScope XT digital slide scanner, and analyzed using ImageScope software (Aperio Technologies, Inc., Vista, CA).

### Endothelial tube formation assay

Cells transfected with miR-NC and miR-126-3p were plated at a density of 3000–4000 cells in a 96-well plate, 48 hours after transfection. Cells were allowed to attach overnight, and matrigel basement matrix (BD Biosciences) was placed over the cells. HUVEC cells (Lonza, Walkersville, MD) were then added on top of the matrigel layer at a density of 60,000 cells per well. The formation of endothelial tubes was observed and photographed over time.

### Genome-wide mRNA expression array

TPC-1 and FTC-133 cells were transfected with miR-126-3p and miR-NC. Three days post-transfection, cells were harvested and total RNA was extracted from cells using TRIzol reagent (Invitrogen, USA). One-hundred fifty nanograms of total RNA were used to perform cDNA reverse transcription, synthesis, amplification, fragmentation, and terminal labeling using GeneChip WT Sense Target Labeling and Control Reagents (Affymetrix, Santa Clara, CA). Approximately 25 ng/μL of cDNA was hybridized to an Affymetrix Human Gene 1.0 ST Array GeneChip. The arrays were washed and stained using the fluidics protocol FS450_0007 procedure on an Affymetrix Fluidics Station 450. The probe intensities were scanned with the GeneChip Scanner 3000. The raw data were normalized and analyzed using Partek Genomic Suite software (Partek, Inc., St. Louis, MO). Variance analysis was used to determine the probe sets that were significantly different between the two groups. The gene list was filtered with a fold-change cutoff of 1.3 and adjusted p < 0.05. The Ingenuity Pathway Analysis (IPA) system (Ingenuity Systems, Redwood City, CA) was used for pathway analysis.

### miRNA-126-3p target predictions

TargetScan 5.1 (http://targetscan.org/) was used to identify potential direct target genes for miR-126-3p.

### Luciferase reporter assay

The 1,573-base pair 3′-UTR of human *ADAM9* was cloned into an empty luciferase reporter vector, pEZX-MT01 (GeneCopoeia, Rockville, MD), generating a wild-type *ADAM9* 3′-UTR luciferase reporter construct (pEZX-ADAM9-3′UTR). The 2,947-base pair 3′-UTR of human *SLC7A5* was also cloned into pEZX-MT01, generating a wild-type *SLC7A5* 3′-UTR luciferase reporter construct (pEZX-SLC7A5-3′UTR). For the dual luciferase assay, FTC-133 cells were plated in triplicate into 24-well plates and co-transfected with 0.25 μg of the reporter construct and 15 pmol of miR-126-3p or miR-NC by using Lipofectamine 2000 (Invitrogen). At 24 hours, the cells were lysed and assayed for both firefly and *Renilla* luciferase activity using the miR Luciferase Assay Kit (GeneCopoeia) on a SpectraMax M5e microplate reader (Molecular Devices, Sunnyvale, CA), according to the manufacturers' instructions.

### Data analysis

The National Cancer Institute’s Cancer Genome Atlas (TCGA) dataset for thyroid cancer was used to determine an association between miR-126-3p expression and disease aggressiveness (data portal, https://tcga-data.nci.nih.gov/tcga/). Normalized intensity values (log 10) for miR-126-3p expression were obtained from 454 papillary thyroid cancer samples. Data are presented as mean ± standard error of the mean. To determine statistical significance, an analysis of variance and t test were used, as appropriate. A p value of less than 0.05 was considered statistically significant.

## Results

### miR-126-3p expression in thyroid cancer is associated with aggressive disease phenotype and tumors with capsular and angioinvasion

**Fig 1:**
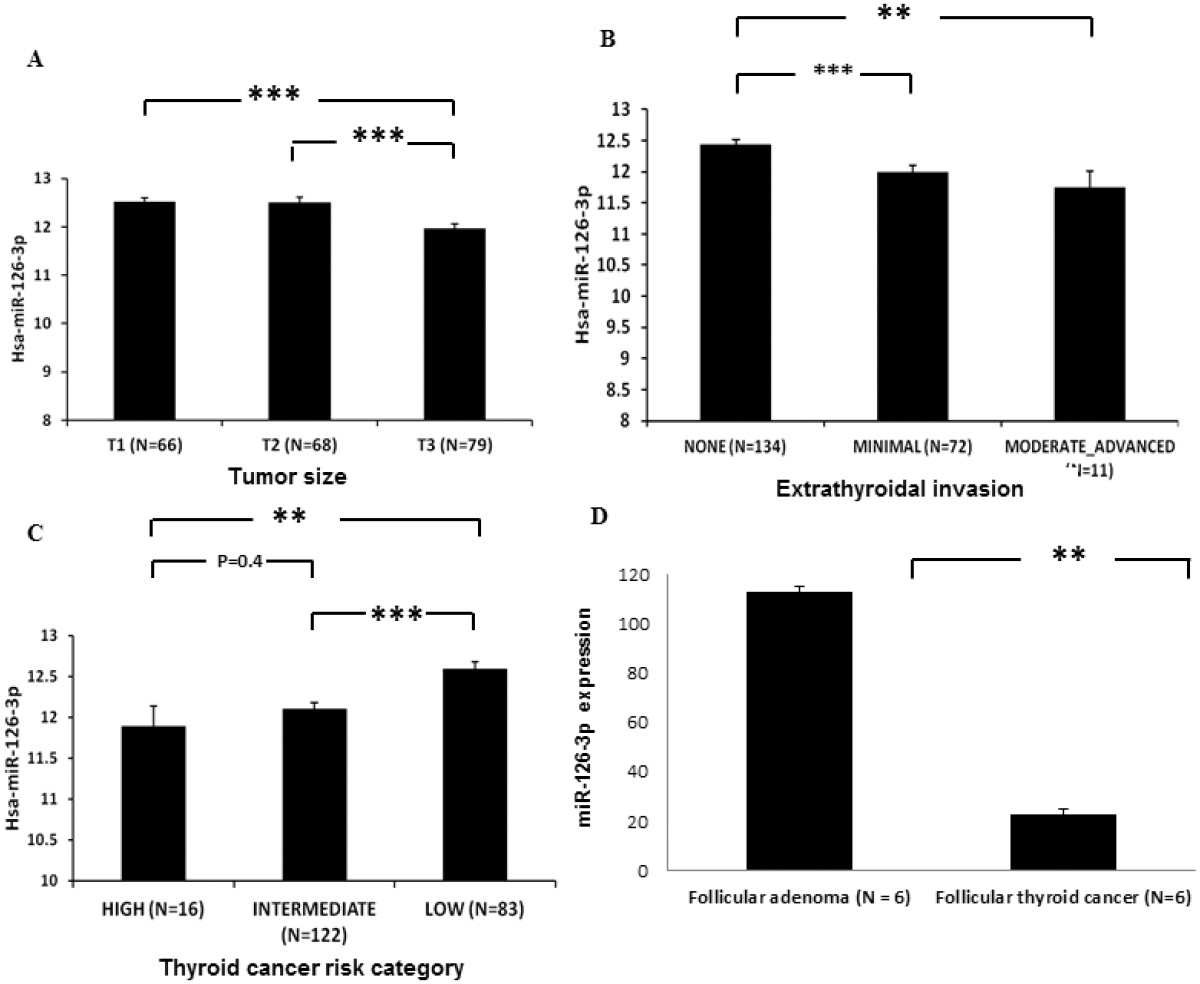
Lower miR-126-3p expression is associated with aggressive thyroid cancer. **(A)** miR-126-3p is significantly lower in larger papillary thyroid cancer (T3 compared to T2 and T1 tumors). Data for TCGA samples with available tumor size data. T1 tumors less than 2cm and not growing outside the thyroid, T2 tumors > 2cm but < 4cm and not growing outside the thyroid, T3 tumors measuring > 4cm or growing outside the thyroid. ** indicates p < 0.01, *** indicates p < 0.001. Y axis is log_10_ normalized expression. **(B)** miR-126-3p expression is significantly lower in papillary thyroid cancer with minimal and moderate extrathyroidal invasion. ** indicates p < 0.01, *** indicates p < 0.001. Y axis is log_10_ normalized expression. **(C)** miR-126-3p expression is significantly lower in high and intermediate MACIS risk papillary thyroid cancer as compared to low MACIS risk tumors. MACIS is a prognostic scoring system used in the TCGA database which is based on the presence of Metastasis, patient Age, Completeness of resection, local Invasion, and tumor Size. ** indicates p < 0.01, *** indicates p < 0.001. Y axis is log_10_ normalized expression. **(D)** miR-126-3p expression is significantly lower in follicular thyroid cancer than follicular thyroid adenoma. All follicular thyroid cancer case had histologic evidence of capsular and vascular invasion and the adenomas did not have any evidence of capsular and vascular invasion. ** indicates p < 0.01. Y axis is 2^-(ΔΔCt).

We had previously identified miR-126-3p to be downregulated in thyroid cancers of follicular cell origin and in tumor types that were difficult-to-diagnose by tumor biopsy examination as capsular invasion and angioinvasion cannot be determine by cytology. Thus, we were interested in determining if miR-126-3p expression was associated with disease aggressiveness and specifically in tumors with capsular invasion and angioinvasion. To investigate this, we used the TCGA dataset for papillary thyroid cancer. We found that miR-126-3p expression was lower in larger primary tumors (Fig 1A), in tumor samples with extrathyroidal invasion (Fig 1B), and in high-risk group thyroid cancer (Fig 1C). Given the association of miR-126-3p with more aggressive papillary thyroid cancer, we next asked if miR-126-3p expression was lower in localized tumors with capsular invasion and angioinvasion using follicular thyroid cancer and adenomas samples for which malignancy is established only by the presence of these two hallmarks of cancer. We found miR-126-3p was significantly lower in localized follicular thyroid cancer as compared to follicular adenomas (Fig 1D). These findings taken together suggest that miR-126-3p may have a significant role in thyroid cancer initiation and or progression.

### miR-126-3p regulates thyroid cancer cell proliferation, colony and spheroid formation, and cellular migration

**Fig 2:**
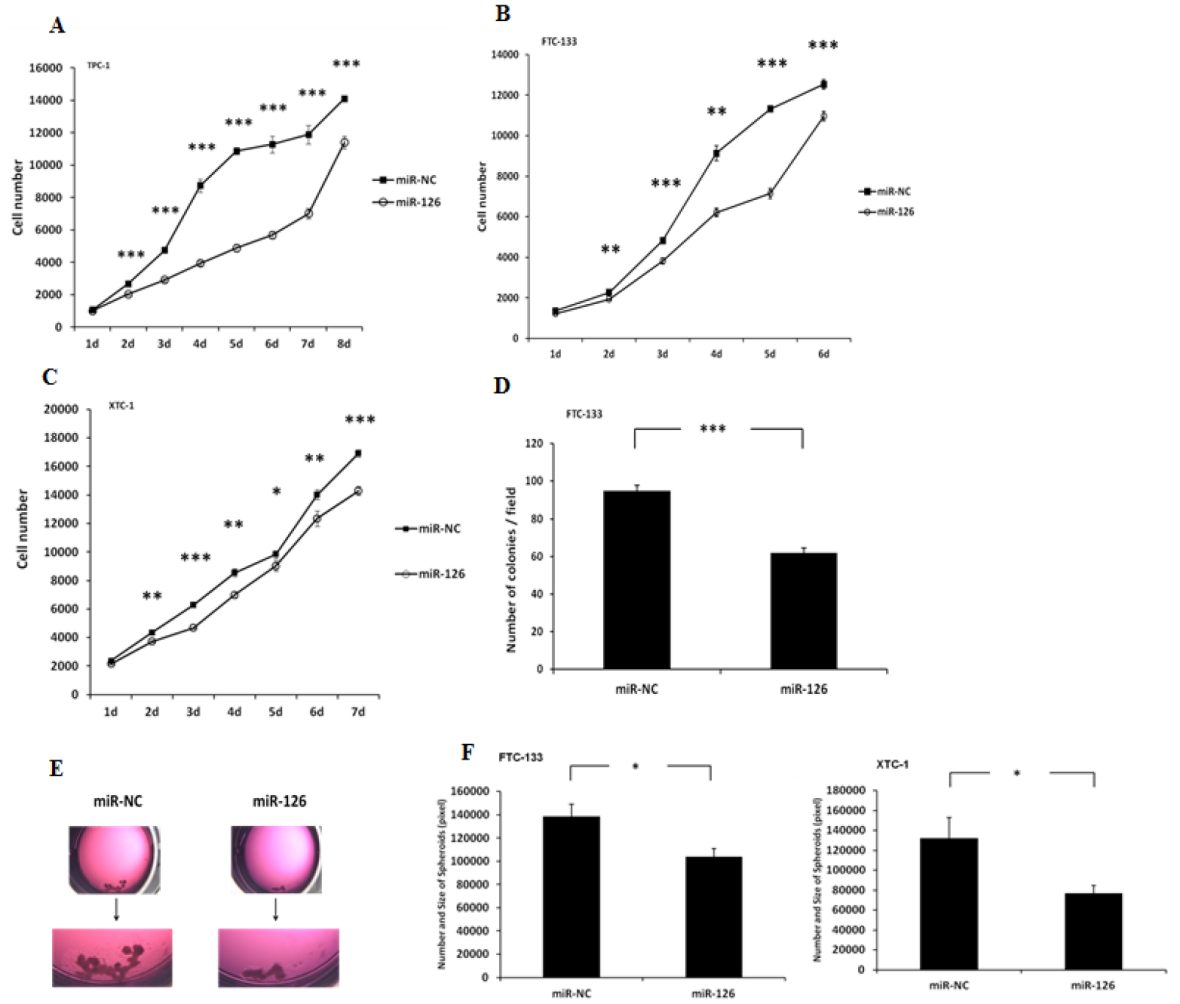
miR-126-3p overexpression inhibits cellular proliferation, and colony and spheroid formation. **(A–C)** Thyroid cancer cell line proliferation with miR-126-3p overexpression. The Y axis represents the cell number. Error bars represent the standard error of the mean (SEM). (* indicates p<0.05; ** indicates p<0.01; *** indicates p<0.001). **(D)** miR-126-3p overexpression inhibits colony formation in thyroid cancer cells. Colony numbers in FTC-133 cell lines. The Y axis represents the number of colonies per field. Error bars represent SEM (*** indicates p<0.001). **(E)** miR-126-3p overexpression decreases the size and number of spheroids. Top panel: representative image of spheroids in culture with miR-126-3p overexpression (FTC-133 cells). Lower panel: Quantification of spheroid differences between XTC-1 and FTC-133 cells with miR-126-3p overexpression. The total area occupied by the spheroids within an image was measured by circumscribing the perimeter of each spheroid, marking the entire area, and calculating the pixel numbers with ImageJ software (National Institutes of Health, Bethesda, MD, USA). The Y axis represents the size and number of the spheroids. Error bars represent SEM (* indicates p<0.05).

Given the association between miR-126-3p expression and aggressive thyroid cancer disease phenotype, we wanted to determine if the function of miR-126-3p in thyroid cancer could mechanistically explain this association. We overexpressed miR-126-3p in three well-characterized and authenticated thyroid cancer cell lines (TPC-1, FTC-133 and XTC-1) using miR-NC as a negative control to determine its effect on cellular proliferation. miR-126-3p overexpression inhibited cell proliferation significantly in TPC-1 and FTC-133 cells at 120 hours, by 52% (p<0.001) and 37% (p<0.001), respectively; however, it inhibited proliferation only by 16% in XTC-1 cells at 168 hours (p<0.001) (Fig 2A, 2B and 2C). A soft agar colony formation assay was performed to evaluate anchorage-independent growth in FTC-133, which is a colony-forming thyroid cancer cell line. We found a significantly lower number of colonies in FTC-133 cell lines overexpressing miR-126-3p (Fig 2D). We also studied the effect of miR-126-3p on thyroid cancer cell tumor spheroid formation. The FTC-133 and XTC-1 cell lines form spheroids when cultured in ultra-low adherent culture flasks, and after transfection with miR-126-3p, the number and size of spheroids were significantly decreased (Fig 2E).

**Fig 3:**
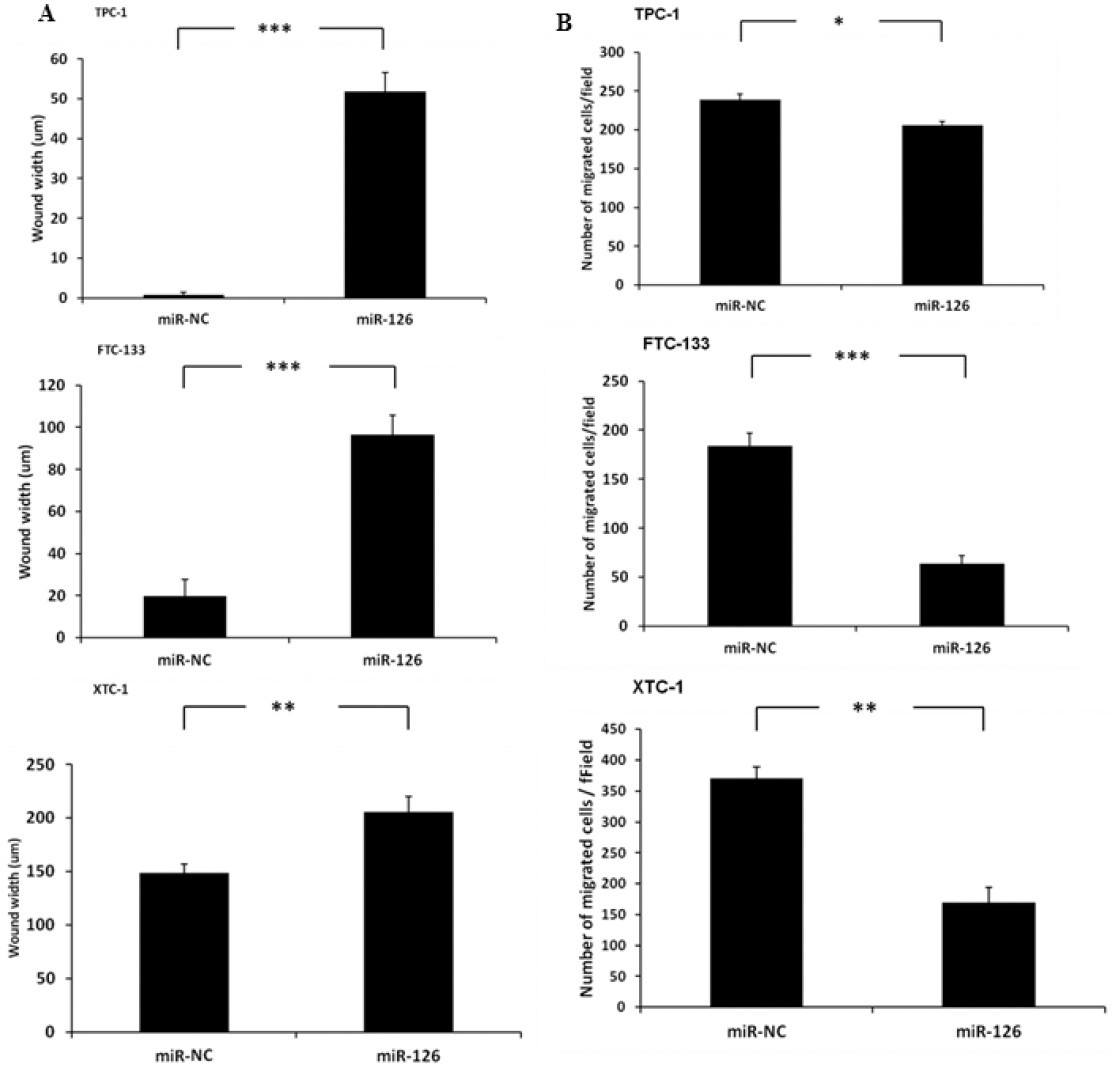
MiR-126-3p overexpression inhibits the migration of thyroid cancer cells. Wound healing assay **(A)** and Boyden chamber assay **(B)** data. MiR-126-3p overexpression significantly decreased wound width closure at 24 hours in all thyroid cancer cell lines studied. The Y axis represents the wound distance. Error bars represent SEM (** indicates p<0.01; *** indicates p<0.001). MiR-126-3p overexpression significantly decreased the number of migrated cells in all thyroid cancer cells in the Boyden chamber assay. The Y axis represents the number of migrated cells per field. Error bars represent SEM (* indicates p<0.05; ** indicates p<0.01; *** indicates p<0.001).

We next determined the effect of miR-126-3p on cellular migration using the scratch wound healing assay. We found that overexpression of miR-126-3p significantly inhibited wound closure in TPC-1 cells (p<0.001), FTC-133 cells (p<0.001), and XTC-1 cells (p<0.01) (Fig 3A). To confirm our findings, we also used a Boyden chamber assay to study the effect of miR-126-3p on cellular migration. We found that overexpression of miR-126-3p also significantly inhibited cell migration (Fig 3B).

### miR-126-3p regulates thyroid tumor growth and metastasis *in vivo*

**Fig 4:**
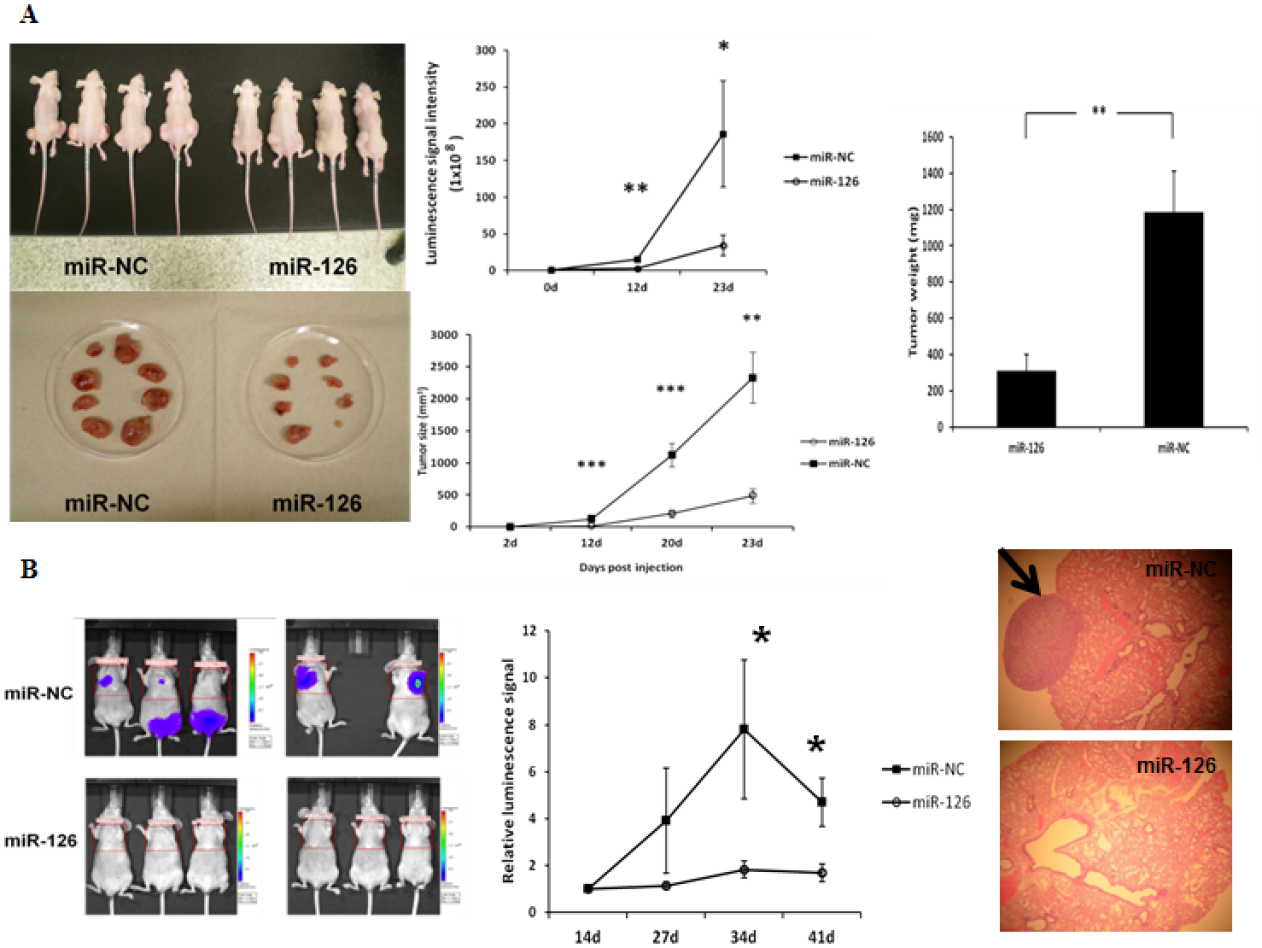
MiR-126-3p overexpression inhibits tumor growth and tumor metastasis *in vivo*. **(A)** Growth of tumor xenografts in nude mice. **Left panel**: representative images of mice xenograft size at autopsy. **Middle and right panel**: tumor luciferase activity and tumor volume measurement, and weight. FTC-133-Luc2 cells transfected with miR-126-3p and miR-NC were inoculated subcutaneously in the flanks of athymic nude mice. **(B)** Tumor metastasis. **Left panel**: Representative images of mice with metastases showing luminescence signal. **Middle panel**: Quantification of luminescence signal intensity differences between miR-126-3p and miR-NC. FTC-133-Luc2 cells transfected with miR-126-3p and miR-NC were injected into athymic nude mice via the tail vein, and the mice were imaged with a Xenogen IVIS 100 system. The relative luminescence signal of each mouse is calculated as the ratio of original signal to the signal taken 14 days post-injection. The images shown here were taken 7 weeks after vein injection of tumor cells. Error bars represent SEM (* indicates p<0.05; ** indicates p<0.01; *** indicates p<0.001). All animal experiments were repeated twice. **Right panel**: A representative microscopic image (hematoxylin and eosin [H&E] staining) of metastatic lung tumor induced by FTC-133-Luc2 cells transfected with miR-NC and an H&E-stained section of metastatic lung tumor induced by FTC-133-Luc2 cells transfected with miR-126-3p.

Given our *in vitro* data, we wanted to evaluate the effect of miR-126-3p on tumor growth *in vivo* and determine whether transiently elevated levels of miR-126-3p could have a sustained, long-term phenotypic effect. We found that tumor xenografts derived from FTC-133-*luc2* cells transfected with miR-126-3p were significantly smaller and weighed less than tumor xenografts from the miR-NC group (p<0.01) (Fig 4A). Because one of the most dramatic effects of miR-126-3p overexpression *in vitro* was on cellular migration, we next determined if miR-126-3p regulated metastasis *in vivo*. To determine whether overexpression of miR-126-3p could inhibit tumor metastasis *in vivo*, FTC-133-*luc2* cells transfected with miR-126-3p and miR-NC were injected into athymic nude mice via the tail vein, and the mice were imaged every week. We found that overexpression of miR-126-3p dramatically suppressed lung metastasis in this model (Fig 4B).

### miR-126-3p regulates ADAM9 and SLC7A5 expression in thyroid cancer cells

Given that miR-126-3p had an effect on cell proliferation, migration and metastasis *in vitro* and *in vivo*, we were interested in determining its target pathway(s) and gene(s). We used two approaches to determine candidate miR-126-3p targets: (1) genome-wide expression analysis with overexpression of miR-126-3p and (2) a target prediction database analysis.

**Fig 5:**
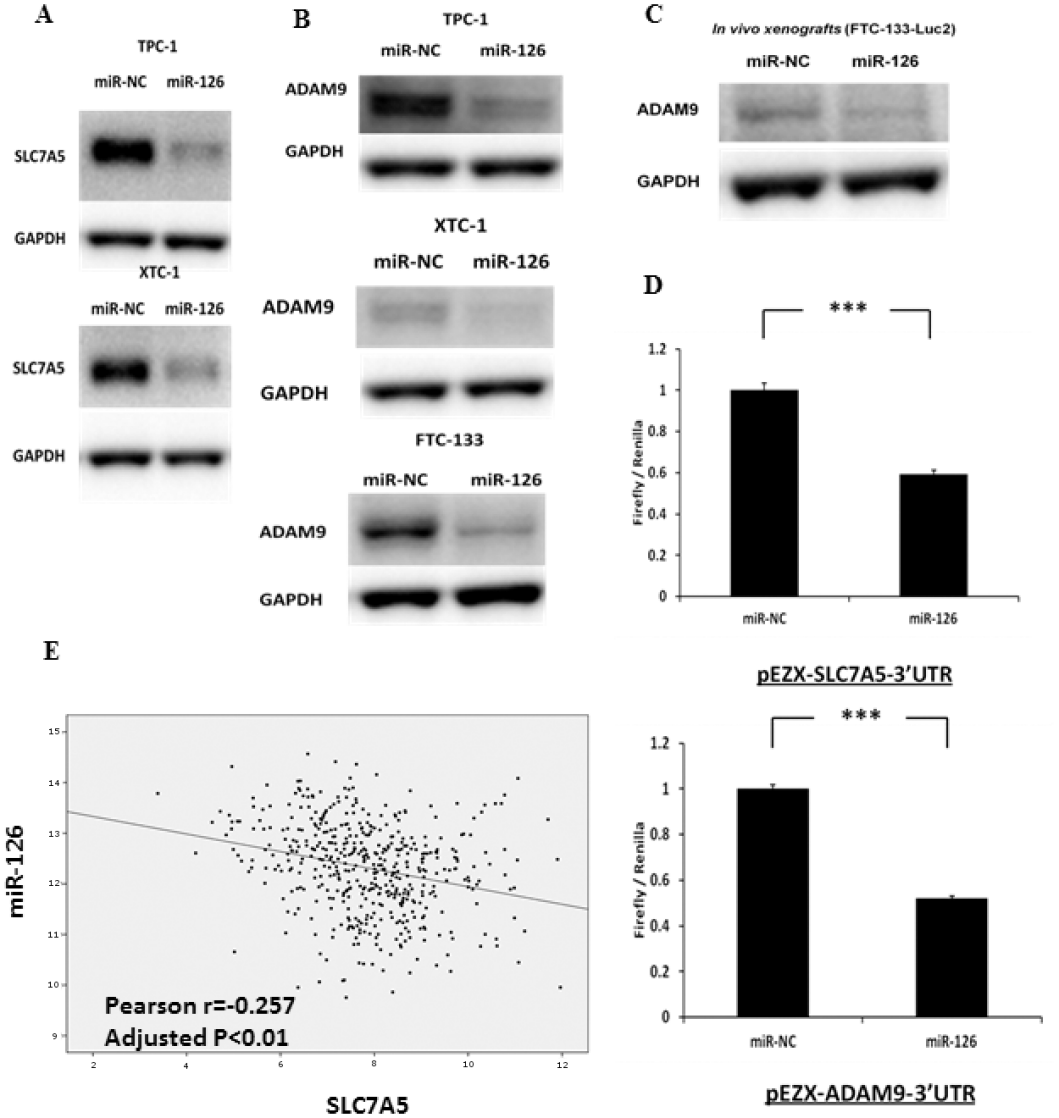
miR-126-3p regulates and directly targets SLC7A5 and ADAM9 protein expression in thyroid cancer cells *in vitro* and *in vivo*. **(A)** Immunoblots of SLC7A5 and GAPDH in TPC-1 and XTC-1 cell lines, which were transfected with either miR-126-3p or miR-NC for 72 hours. The FTC-133 cell line had no detectable protein expression for SLC7A5. **(B)** Immunoblots for ADAM9 and GAPDH in TPC-1, FTC-133 and XTC-1 cell lines, which were transfected with either miR-126-3p or miR-NC for 72 hours *in vitro*. **(C)** Immunoblots for detecting ADAM9 and GAPDH in FTC-133-Luc2 tumor xenografts that had been inoculated subcutaneously into the flanks of athymic nude mice and allowed to develop for 10 days. **(D)** Luciferase activity of pEZX-SLC7A5-3′UTR and pEZX-SLC7A5-3′UTR in FTC-133 cells when co-transfected with miR-126-3p or miR-NC. All luciferase measurements were made in triplicate and readings were performed 24 hours post-transfection. Error bars represent SEM (*** indicates p<0.001). **(E)** The expression level of miR-126-3p is significantly inversely associated with the expression level of *SLC7A5* in 481 papillary thyroid cancer samples from the TCGA dataset. *** indicates p<0.001.

Integrated analyses of the genome-wide gene expression data and target scan prediction revealed 14 genes as targets of miR-126-3p (S1 Table). We then performed a pathway analysis with these 14 genes. The top diseases and biological functions identified by IPA are summarized in S2 Table. Cancer was the top disease category, with four molecules involved in this pathway that were significantly reduced by miR-126-3p overexpression. Furthermore, among the 14 genes, we found that *SLC7A5* had the highest fold changes in both cell lines upon miR-126-3p overexpression, and *ADAM9* had the second highest fold change (2.8-fold) in FTC-133 cells, which were used for both *in vitro* and *in vivo* assays in the present study. Both *ADAM9* and *SLC7A5* play important roles in several cancers and have been shown to be targeted by miR-126-3p [13–16]. Thus, we were interested in determining whether SLC7A5 and ADAM9 protein levels were altered upon miR-126-3p overexpression. We found that miR-126-3p overexpression reduced SLC7A5 protein expression in TPC-1 and XTC-1 cells (Fig 5A) and reduced ADAM9 protein expression in all three cell lines (TPC-1, FTC-133, and XTC-1) (Fig 5B). We also found that miR-126-3p overexpression downregulated ADAM9 protein expression in FTC-133-*luc2* tumor xenografts that had been inoculated subcutaneously into the flanks of athymic nude mice and allowed to develop for 10 days (Fig 5C). Given these results, we next determined whether *ADAM9* and/or *SLC7A5* were direct targets of miR-126-3p. We performed luciferase assays on FTC-133 cells co-transfected with pEZX-ADAM9-3′UTR (vector with 3′-UTR of *ADAM9*) and miR-126-3p or miR-NC. We found that miR-126-3p overexpression significantly decreased luciferase activity as compared to the negative control (Fig 5D), suggesting that miR-126-3p directly targets the 3′-UTR region of *ADAM9* and *SLC7A5* in thyroid cancer cells. We next analyzed whether there was an association between miR-126-3p expression and ADAM9 and SLC7A5 mRNA expression in the TCGA papillary thyroid cancer dataset, and found a significant inverse association with SLC7A5 mRNA expression (r = −0.257, p<0.01) but not with ADAM9 mRNA expression (Fig 5E).

### miR-126-3p reduces VEGF secretion and endothelial tube formation

**Fig 6:**
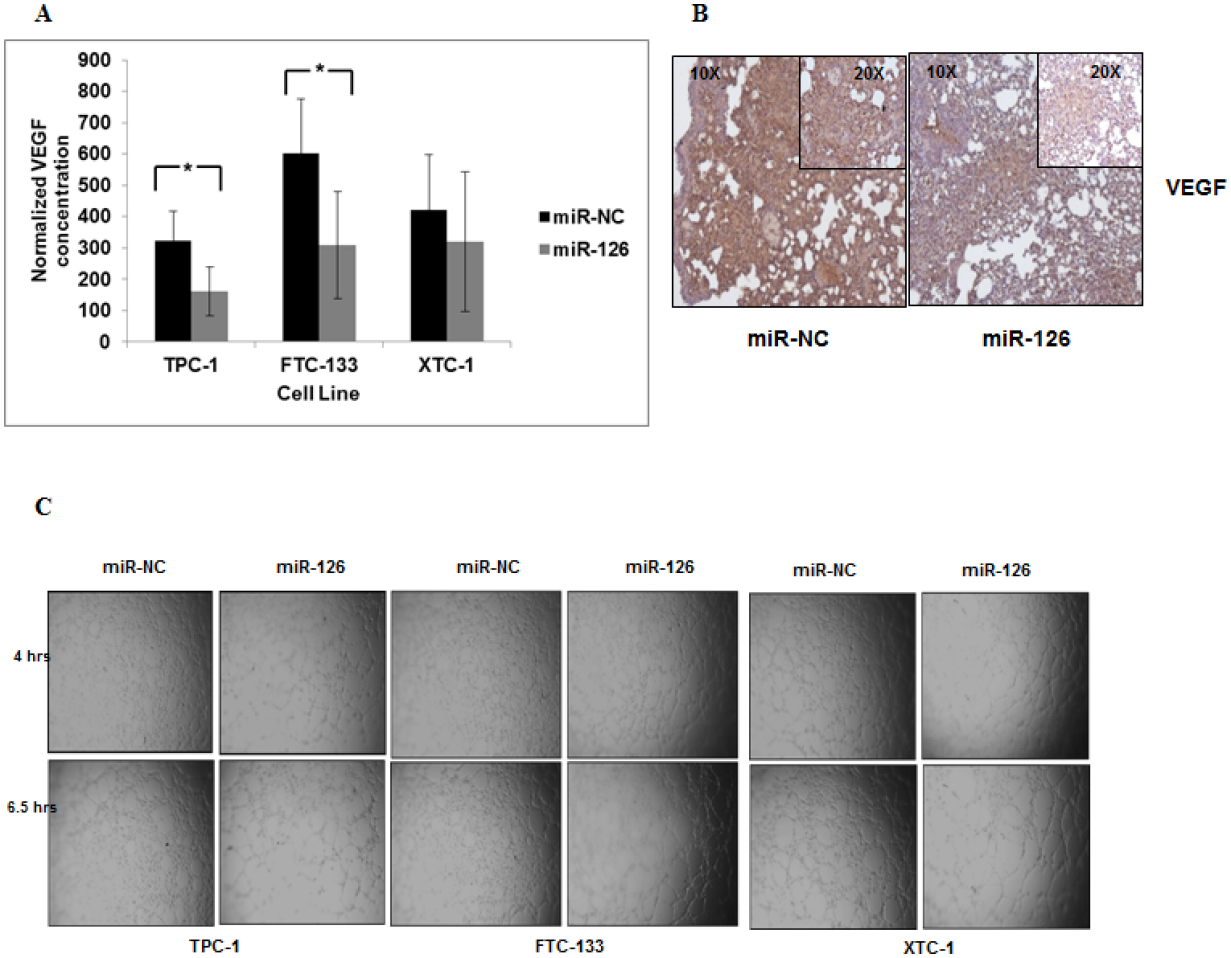
miR-126-3p reduces VEGF secretion and endothelial tube formation. **(A)** miR-126-3p overexpression significantly reduced secreted VEGF levels in TPC-1 and FTC-133 cell lines but not XTC-1 cells. * p < 0.5. VEGF levels were also normalized to total protein. Culture media was harvested from TPC-1, FTC-133 and XTC-1 cells transfected with miR-NC and miR-126-3p after 72 hours of transfection. **(B)** Expression of VEGF protein in lung tumor metastases was decreased in mice injected with FTC-133 Luc cells transfected with miR-126-3p as compared to miR-NC. **(C)** Endothelial cell tube formation is decreased with miR-126-3p overexpression. All experiments were repeated at least three times.

Given that we found miR-126-3p expression is lower in localized follicular thyroid cancer with capsular and vascular invasion, and miR-126-3p has been reported to regulate angiogenesis and target *VEGF*, we next determined whether miR-126-3p regulates angiogenesis in thyroid cancer cells [17–20]. We found that miR-126-3p overexpression significantly decreased VEGF secretion in two of three thyroid cancer cells *in vitro* (Fig 6A). One of the principal determinants for successful tumor growth is the ability to recruit new blood vessels. Thus, we analyzed the VEGF protein expression level in the lung metastatic tumors from the *in vivo* studies and endothelial tube formation by using the HUVEC assay *in vitro*. We found that miR-126-3p overexpression decreased VEGF protein expression in metastatic tumor xenografts cells and reduced endothelial cell tube formation (Fig 6B and 6C). These findings are consistent with our results of reduced miR-126-3p expression in localized follicular thyroid cancer with angioinvasion.

## Discussion

In the present study, we show that decreased miR-126-3p expression is associated with clinically more aggressive papillary thyroid cancer characterized by larger primary tumor size, local invasion and high-risk cancers for recurrence, as well as in follicular thyroid cancers as compared to follicular adenomas. Given these significant associations with more aggressive human thyroid cancer, we sought to understand the mechanism by which miR-126-3p functionally affects thyroid cancer phenotype. We found ectopic overexpression of miR-126-3p significantly inhibited thyroid cancer cell proliferation, colony formation, tumor spheroid formation and migration, and VEGF secretion in thyroid cancer cell lines *in vitro*, and significantly inhibited tumor growth and metastasis *in vivo*. These findings suggest that miR-126-3p functions as a tumor suppressor in thyroid cancer. We also found that miR-126-3p targets genes involved in cancer, and directly regulates *SLC7A5* and *ADAM9* expression. This suggests that *SLC7A5* and *ADAM9* are two target genes among others that mediate the tumor suppressive effects of miR-126-3p on growth and migration in thyroid cancer cells.

The sequence encoding miR-126-3p is located at intron 7 of the *EGFL7* gene, and recently Saito and associates found that the expression of miR-126-3p may be epigenetically regulated with the *EGFL7* gene [21]. Downregulation of miR-126-3p expression has been found in colorectal cancer, cervical cancer, small cell lung cancer, breast cancer, and gastric cancer [22–26]. Feng et al. found that miR-126-3p inhibited tumor growth and metastasis *in vitro* and *in vivo* in human gastric cancer by directly targeting Crk [26]. MiR-126-3p was also reported to suppress breast cancer cell growth by targeting insulin receptor substrate-1 (IRS-1) [25], and inhibit proliferation of small cell lung cancer cells by targeting *SLC7A5* [16]. Guo et al. showed that miR-126-3p suppressed colon cancer cell growth by directly targeting the 3′-UTR of p85beta [27]. Hamada et al. found that miR-126-3p plays a role as a tumor suppressor in pancreatic cancer cells by targeting *ADAM9* [28]. Tumor spheroid model have been used to enrich for cancer stem cells. We found that miR-126-3p overexpression reduced the number and size of the tumor spheroid suggesting it may also reduce cancer stem cell population. These findings suggest that miR-126-3p has multiple target genes, which could mediate its effect on cancer growth and metastasis.

*SLC7A5* encodes a large, transmembrane neutral amino acid transporter that transports thyroid hormones as secondary substrates [29]. Expression of *SLC7A5* has been detected in a variety of tumor cells, including thyroid cancer, teratocarcinoma, bladder cancer, lung cancer, melanoma, hemangiopericytoma, and uterine cervical cancer [29–31]. *SLC7A5* is overexpressed in various types of cancer, including thyroid cancer, and various levels of SLC7A5 overexpression have been associated with high-grade malignancies and poor prognoses [32–35]. Inhibition of SLC7A5 protein expression reduced cancer cell proliferation in some types of cancer [36–39]. Recently, Miko et al. also showed that miR-126-3p inhibits cellular proliferation of small cell lung cancer by directly targeting *SLC7A5* [16]. In the present study, we show that overexpression of miR-126-3p significantly inhibits proliferation of thyroid cancer cells and reduces the expression of SLC7A5 protein in thyroid cancer cells, and that miR-126-3p and *SLC7A* expression is inversely correlated in TCGA’s thyroid cancer sample database.

*ADAM9* is a member of the ADAM (a disintegrin and metalloprotease domain) family, which plays an essential role in the protein ectodomain shedding of membrane-bound molecules [40]. Knocking out *ADAM9* in mice does not lead to an obvious phenotype during development or in adult mice [41]. Overexpression of *ADAM9* has been found in thyroid cancer and other cancers, including breast cancer, hepatocellular carcinoma, pancreatic cancer, prostate cancer, and melanoma [35,40,42–47]. Previous studies have shown that *ADAM9* affects tumor migration, invasion, and metastasis by modulating tumor cell adhesion [48,49]. Hamada et al. found that miR-126-3p inhibits pancreatic cancer cell migration and invasion by directly targeting *ADAM9* [28]. In the present study, we show overexpression of miR-126-3p significantly inhibits thyroid cancer cell migration and metastasis, and that the protein expression level of ADAM9 is reduced in thyroid cancer cell lines in which miR-126-3p is overexpressed. We also show that miR-126-3p regulates the 3′-UTR of *ADAM9*, using a luciferase reporter assay. Taken together, these data indicate that *ADAM9* likely mediates the effects of miR-126-3p on cellular migration and metastasis.

We performed an integrated analysis of a genome-wide expression analysis with overexpressed miR-126-3p and a target scan prediction, and found 14 candidate genes targeted by miR-126-3p. We validated *SLC7A5* and *ADAM9* as direct targets of miR-126-3p because they were the most downregulated genes, and each has been shown to regulated cellular proliferation and migration/invasion, respectively. Obviously, since the top disease category indicated by our IPA was cancer, it is possible that other genes may also be targets of miR-126-3p and may influence thyroid cancer progression. Other genes we found to be altered by miR-126-3p expression include *PIK3R2* and *CAMSAP1*, which have also been implicated in cancer biology [50,51].

To our knowledge, this is the first study to demonstrate that miR-126-3p has a tumor-suppressive function in thyroid cancer, that it is associated with aggressive disease, that it regulates angiogenesis and VEGF secretion, and that it directly regulates SLC7A5 and ADAM9 protein expression via regulation of the 3′-UTR of these two genes.

## Supporting Information

S1 Table (DOC)

S2 Table (DOC)
