# Supplementary material for: miR-126-3p Inhibits Thyroid Cancer Cell Growth and Metastasis, and Is Associated with Aggressive Thyroid Cancer
Source: PLoS One. 2015 Aug 5;10(8):e0130496. doi: 10.1371/journal.pone.0130496 (PMC4526518; doi:10.1371/journal.pone.0130496)
Supplement: S1 Table — (DOC) [file pone.0130496.s001.doc]

**Supplemental Table 1.** Mir-126-3p candidate target genes

|  | **FTC-133 cell**  **(miR-NC vs. miR-126-3p)** | **TPC-1 cell**  **(miR-NC vs. miR-126-3p)** |
| --- | --- | --- |

| **Gene Symbol** | **Fold-Change** | **p-value** | **Fold-Change** | **p-value** |
| --- | --- | --- | --- | --- |
| *SLC7A5* | 3.13 | 5.49E-07 | 4.99 | 4.12E-05 |
| *ADAM9* | 2.80 | 1.63E-08 | 1.70 | 6.13E-09 |
| *KIAA1715* | 2.78 | 5.67E-07 | 2.20 | 6.24E-08 |
| *PLXNB2* | 2.35 | 3.10E-06 | 1.70 | 1.23E-05 |
| *B4GALT4* | 2.21 | 6.13E-06 | 1.58 | 1.34E-04 |
| *PMM1* | 2.02 | 1.20E-05 | 2.12 | 5.83E-07 |
| *PIK3R2* | 1.94 | 2.31E-06 | 1.90 | 1.18E-04 |
| *MMGT1* | 1.91 | 4.90E-06 | 1.43 | 4.18E-05 |
| *SLC41A2* | 1.65 | 3.52E-05 | 1.32 | 3.56E-05 |
| *PTPN9* | 1.64 | 5.00E-06 | 1.39 | 1.19E-06 |
| *CAMSAP1* | 1.60 | 2.31E-06 | 1.53 | 4.48E-04 |
| *SLC15A4* | 1.53 | 6.62E-05 | 1.32 | 4.25E-04 |
| *GOLPH3* | 1.52 | 4.05E-06 | 1.36 | 6.19E-06 |
| *EFHD2* | 1.49 | 6.51E-05 | 2.16 | 1.19E-05 |
