# Supplementary material for: miR-126-3p Inhibits Thyroid Cancer Cell Growth and Metastasis, and Is Associated with Aggressive Thyroid Cancer
Source: PLoS One. 2015 Aug 5;10(8):e0130496. doi: 10.1371/journal.pone.0130496 (PMC4526518; doi:10.1371/journal.pone.0130496)
Supplement: S2 Table — (DOC) [file pone.0130496.s002.doc]

**Supplemental Table 2.** Top Diseases and Disorders revealed by Ingenuity pathway analysis

**________________________________________________________________**

**Name p-value # Molecules**

Cancer 8.06E-04 - 3.35E-02 4

Hereditary Disorder 8.06E-04 - 5.63E-03 3

Neurological Disease 8.06E-04 - 3.41E-02 5
